# Differences in the inflammatory response among hospitalized patients with distinct variants of SARS-CoV-2

**DOI:** 10.3389/fimmu.2023.1267991

**Published:** 2023-10-16

**Authors:** Jose-Reynaldo Homen-Fernandez, Adrián Valls, Ana García, Noemí Cabello, Isabel Ortega, Eva Orviz, Carlos Foncubierta, Mercedes Martínez, Vicente Estrada

**Affiliations:** ^1^ Servicio de Enfermedades Infecciosas, Instituto de Investigación Sanitaria del Hospital Clínico San Carlos (IdISSC), Hospital Clínico San Carlos, Madrid, Spain; ^2^ Servicio de Análisis Clínicos, Instituto de Medicina del Laboratorio, Hospital Clínico San Carlos, Madrid, Spain; ^3^ Centro de Investigación Biomédica en Red de Enfermedades Infecciosas (CIBERINFEC), Instituto de Salud Carlos III, Madrid, Spain; ^4^ Centro Sanitario Sandoval, Hospital Clínico San Carlos, Madrid, Spain; ^5^ Hospital Clínico San Carlos, IdISSC, Unidad de Apoyo Metodológico a la Investigación y departamento preventivo, Madrid, Spain

**Keywords:** COVID-19 variants, omicron, delta, alpha, inflammation, SARS-CoV2 (COVID- 19), Spain

## Abstract

The SARS-CoV-2 variants demonstrate diverse transmission patterns, modifications in infectivity, and immune response. Changes in disease manifestation may be attributed to vaccination and the virus’s reduced capacity to induce inflammation.

**Objectives:**

To investigate the relationship between the inflammatory response and the characteristics of COVID-19 across successive waves.

**Methods:**

A retrospective cross-sectional study was conducted to evaluate sociodemographic, clinical, and laboratory data of Alpha (G1), Delta (G2), and Omicron (G3) variants.

**Results:**

A total of 300 patients from a hospital in Madrid, Spain, were included. The groups exhibited similar sociodemographic and baseline characteristics. The Alpha variant predominantly affected younger patients, while the Omicron variant affected patients with a higher prevalence of comorbidities. The Alpha group had the lowest vaccination rate compared to the highest rate in the Omicron group. The Alpha group received a higher proportion of tocilizumab compared to the other groups. Despite these differences, the severity scores were similar among the three variants. Regarding laboratory parameters, differences were observed in haemoglobin, D-dimer, alkaline phosphatase, and potassium levels. The Omicron variant showed higher D-dimer levels (p=0.04). In the multivariate analysis, differences in leukocyte count, haemoglobin, alkaline phosphatase, and potassium levels were consistently observed among patients from different waves. Omicron exhibited a higher absolute leukocyte count than the Alpha variant (p=0.003).

**Conclusion:**

No significant differences were found in inflammation biomarkers among the three variants. Furthermore, there were no significant disparities in mortality or disease severity. The level of inflammatory response in patients may be determined by the severity of COVID-19, rather than the specific viral variant.

## 1 Introduction

Since the first identification of the Variant of Concern (VOC), it has been established that structural modifications can alter the course of infection (1) and emerging variants have been closely monitored with the objective of promptly detecting genetic alterations that may impact transmissibility, disease severity, immunogenicity, drug response, and other features (2). The World Health Organization (WHO) has identified five VOCs: Alpha (September 2020), Beta (May 2020), Gamma (November 2020), Delta (October 2020), and Omicron (November 2021) (3, 4).

In Spain, epidemiological surveillance has been conducted since 2021 by sequencing a random sample of cases in each community and releasing information on a biweekly basis through the Spanish Surveillance System (SiViEs) (5). The report dated May 17th, 2022 includes the percentage of each variant among all sequenced samples for each week, where the prevalence of three of the five WHO-identified VOCs - Alpha, Delta, and Omicron - is evident (Annex 1) (6).

These VOCs have exhibited distinct patterns of transmission, disease severity, and immune evasion compared to earlier Wuhan SARS-CoV-2 (7, 8). From the Alpha variant, changes in the genetic code were identified, which lead to structural changes (9, 10) and are associated with increased cellular infectivity (11, 12), decreased neutralizing capacity of vaccine-induced antibodies (13–15), and failure in the detection of some diagnostic tests (16, 17). From the Delta variant, at least nine changes were identified in the spike protein that increase interaction with the ACE2 receptor, thereby enhancing virus infectivity. These changes also reduce the action of neutralizing antibodies and confer a shorter incubation period of 2 to 3 days (17). As for the Omicron variant, at least 60 changes have been identified, including changes in the spike protein that confer certain immune escape by reducing binding to some antibodies identified as neutralizing in previous variants (18). These changes also increase the binding affinity to ACE2, resulting in a higher transmission rate, which has been linked to disease progression (18–21). Although the exact mechanisms behind these disparities are still being researched, the inflammatory response and clinical manifestations of COVID-19 may also differ among these variants.

The severity of the disease’s clinical manifestation is ascertained by the extent of lung involvement, which results in varying degrees of hypoxia and respiratory failure. Inflammation is a critical element of this illness, and a heightened inflammatory reaction is linked with a poor prognosis. Therefore, employing inflammation biomarkers in the management of COVID-19 patients has become widespread during the pandemic. The improved prognosis of later waves of the disease is related mainly to the protection exerted by vaccination and greater knowledge of the disease and its management, and it might be related to the virus’s reduced capacity to provoke a systemic inflammatory response.

We hypothesize that the Wuhan SARS-Cov2 strain may elicit a more severe systemic inflammatory response compared to the more recent variants. Although these newer variants have a higher transmission rate, they Might generate a lower inflammatory response, particularly at the pulmonary level. This effect could be attributed to changes in the affinity of the variants for ACE2 receptors at the pulmonary level. The current study aims to investigate the relationship between the inflammatory response and COVID-19’s clinical characteristics across successive waves.

## 2 Patients and methods

### 2.1 Study design and study population

This is a retrospective cross-sectional study of patients hospitalised for COVID-19 between 2021 and 2022 at the Hospital Clínico San Carlos in Madrid, Spain. By evaluating the findings of the SIVIEs, it has been determined that there are three distinct periods in which the predominant VOC fluctuates and surpasses 80% prevalence. Consequently, a decision was made to compare the patients corresponding to these specific periods. To avoid overlaps between groups, intermediate periods were considered, selecting Group 1 (G1) from 15/03/2021 to 19/04/2021 where the predominant VOC was Alpha, Group 2 (G2) from 02/08/2021 to 12/12/2021 predominantly Delta and Group 3 (G3) from 17/01/2022 to 25/04/2022 predominantly Omicron.

The first 100 admissions that met the inclusion criteria and none of the exclusion criteria in three periods were correlatively selected. The inclusion criterion was hospital admission with microbiological confirmation of SARS-CoV2 infection by PCR and the exclusion criterion was that the main reason for hospitalization was related to another condition or disease of the patient or only for preventive isolation.

Given the exploratory nature of the study, the sample size was arbitrarily set at 100 patients per group.

### 2.2 Variables

Sociodemographic and clinical characteristics were collected from the hospital history: age, sex, comorbidities, smoking or alcohol use, vaccination for COVID-19, date of onset of symptoms, duration of hospitalization, need for ICU, exitus or discharge and treatment received for COVID-19.

We also documented the analytical parameters, markers of inflammation and organ damage obtained from the database of the clinical analysis department of the first blood test performed on patients in the emergency room: Haemoglobin, platelets, total leukocytes, absolute neutrophils, absolute lymphocytes, absolute monocytes, prothrombin time, INR prothrombin time, aPTT, D-dimer, Fibrinogen, Glycaemia, Total Bilirubin, Creatinine, estimated glomerular filtration rates (eGFR), Urea, ALT, AST, Alkaline Phosphatase, GGT, LDH, Ck, Sodium, Potassium, Troponin I, Nt-proBNP, CRP (C-Reactive Protein), Procalcitonin, Ferritin.

### 2.3 Statistical analysis

Continuous and ordinal variables were described by the mean and standard deviation or median and interquartile range as appropriate. Categorical variables were described as counts and percentage. For all comparisons between groups, the χ² test, or the Fisher exact test whenever necessary, were applied for categorical variables. Continuous variables were tested for normality with the Kolmogorov-Smirnov test and then compared between groups using a t test, Mann-Whitney test, ANOVA, Kruskal-Wallis test when appropriate. All tests were performed as 2-sided and P values <0.05 were considered significant. To adjust for potential confounding variables, we performed a linear regression analysis adjusting for severity (measured in the WHO scale) and age. We performed a correction of the p values according to the method described by Benjamini and Hochberg (BH) controlling for the false discovery rate. The statistical analysis was performed in R version 4.3.1 and RStudio.

## 3 Results

### 3.1 Baseline characteristics

**Figure 1:**
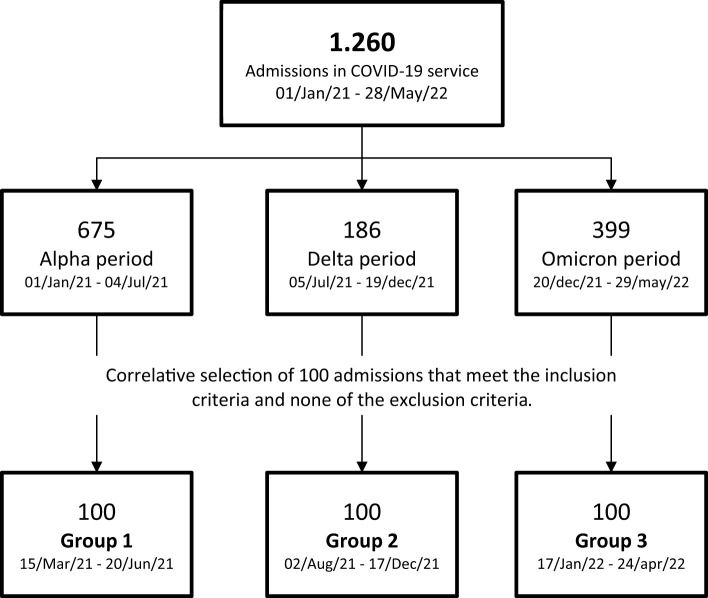
Patient inclusion route: Between January 1st, 2021 and May 28th, 2022, a total of 1260 patients were admitted to the dedicated COVID-19 ward. Throughout this specified timeframe, 100 patients were selected from each corresponding period representing the alpha, delta, and omicron variants. A total of 300 patients were included in the study.

Between January 1st, 2021 and May 28th, 2022, a total of 1260 patients were admitted to the COVID-19 ward. During this period, 100 patients were selected from each corresponding period of the alpha, delta, and omicron variants, resulting in a total of 300 included patients (**Figure 1**). The sociodemographic and baseline characteristics were similar across the three groups, with significant differences in age. Compared to G1, G2 median age was 7.5 years older 7.5 years higher (p=0.009), and G3 was a median 10 years older (p=0.001). Overall, G3 exhibited a higher prevalence of comorbidities compared to G2 and G1. Specifically, G3 had a 6% higher prevalence of chronic kidney disease (CKD) compared to G2, and a 10% higher prevalence compared to G1 (p=0.041). Furthermore, there was a greater incidence of congestive heart failure (CHF) in G3, being 6% and 8% higher than in G1 and G2, respectively (p=0.032). Lastly, G3 displayed a higher proportion of individuals with a history of cancer (p=0.007) (**Table 1**).

**Table 1:** Demographics and baseline characteristics.

Characteristic	G1 N=100	G2 N=100	G3 N=100	P value
Age ― Median (IQR) - yr.	73.5 [62.8;80.0]	81.0 [59.8;88.2]	83.5 [75.8;89.0]	**<0.001**
Sex ― No. (%)				0,886
Male	53 (53)	53 (53)	56 (56)	
Female	47 (47)	47 (47)	44 (44)	
Coexisting condition ― No. (%)				
Hypertension	56 (56)	57 (57)	68 (68)	0,157
Dyslipidaemia	48 (48)	39 (39)	44 (44)	0,438
Diabetes	34 (34)	28 (28)	28 (28)	0,565
Obesity	17 (17)	17 (17)	15 (15)	0,907
Coronary artery disease	9 (9)	4 (4)	10 (10)	0,232
COPD	3 (3)	8 (8)	9 (9)	0,190
CKD	4 (4)	8 (8)	14 (14)	**0,041**
Asthma	6 (6)	3 (3)	8 (8)	0,306
Congestive heart failure	4 (4)	2 (2)	10 (10)	**0,032**
History of cancer	7 (7)	6 (6)	19 (19)	**0.007**
Smoking or alcohol use ― No. (%)				
Former smoker	21 (21.0)	18 (18.0)	29 (29.0)	0,158
Smoker	9 (9.00)	8 (8.00)	11 (11.0)	0,759
alcohol	3 (3.00)	2 (2.00)	4 (4.00)	0,912

IQR, interquartile range; yr, year; COPD, Chronic obstructive pulmonary disease; CKD, Chronic Kidney Disease. Bold values refers to values with a statistically significant p-value (p<0.05).

### 3.2 Clinical presentation of COVID-19

The characteristics associated with COVID-19 are presented in **Table 2**. The median time to admission was 5 days in all three groups. The highest severity score recorded during admission for all three groups was WHO category 4. The majority of patients exhibited a radiological pattern of bilateral pneumonia in the initial imaging test conducted upon admission, with nearly 80% observed in G1, which had the highest percentage of patients.

**Table 2:** Characteristics of COVID-19.

Characteristic	G1 N=100	G2 N=100	G3 N=100	P value
Days until admission ― Median (IQR)	5.00 (3.00;9.00)	5.00 (3.00;7.00)		0.41
Severity OMS ― No. (%)				0.275
3	17 (17.0)	16 (16.0)	19 (19.0)	
4	64 (64.0)	71 (71.0)	64 (64.0)	
5	8 (8.0)	2 (2.0)	3 (3.0)	
6	3 (3.0)	1 (1.0)	0	
8	8 (8.0)	10 (10.0)	14 (14.0)	
Hospitalization duration ― Median (IQR) - Days	9.00 (7.00;11.0)	8.00 (5.00;11.0)	7.00 (5.00;10.0)	**0.077**
ICU admission ― No. (%)	6 (6.00)	5 (5.00)	2 (2.00)	0.453
Death ― No. (%)	8 (8.00)	10 (10.0)	14 (14.0)	0.376
Chest radiography pattern ― No. (%)				<0.001
Bilateral pneumonia	79 (79.0)	61 (61.6)	48 (48.0)	
Localized pneumonia	9 (9.00)	7 (7.07)	12 (12.0)	
Other alteration	2 (2.00)	11 (11.1)	19 (19.0)	
Normal	10 (10.0)	20 (20.2)	21 (21.0)	
COVID-19 vaccination dose				
First ― No. (%)	19 (19.0)	77 (77.0)	84 (84.0)	**<0.001**
Second — no./total no. (%)	8/19 (42.1)	67 / 77 (87.0)	83 / 84 (100)	**<0.001**
Third — no./total no. (%)	1/8 (12.5)	4 / 67 (5.97)	71 / 71 (85.5)	**<0.001**
Treatment ― No. (%)				
Dexamethasone	92 (92.0)	87 (87.0)	78 (78.0)	**0.017**
Metilprednisone	6 (6.00)	5 (5.00)	9 (9.00)	0.498
Remdesivir 3 days	10 (10.0)	18 (18.0)	22 (22.0)	0.068
Remdesivir 5 days	14 (14.0)	11 (11.0)	16 (16.0)	0.585
Tocilizumab	16 (16.0)	3 (3.00)	3 (3.00)	**<0.001**
Prophylactic anticoagulation	86 (86.0)	88 (88.0)	55 (55.0)	**<0.001**
Antimicrobial	47 (47.0)	44 (44.0)	41 (41.0)	0.694

IQR, interquartile range. Bold values refers to values with a statistically significant p-value (p<0.05).

Regarding vaccination, G3 demonstrated the highest percentage of vaccinated individuals, reaching nearly 85%, which was proportionally similar to G2 with an approximate vaccination rate of 80%. In contrast, in G1, less than 20% of individuals had received vaccinations, and there was a statistically significant absolute difference of 56% (p<0.001) between G1 and G2, as well as 65% (p<0.001) between G1 and G3.

In terms of COVID-19 treatment, the majority of patients received dexamethasone, followed by Remdesivir. Notably, G1 received 13% more tocilizumab compared to G2 and G3, and this difference was statistically significant (p=0.006). G3, on the other hand, received 33% less anticoagulant therapy than both G1 and G2, and this difference was also statistically significant (p<0.001). Additionally, it is worth mentioning that over 40% of participants in all three groups had received some form of antimicrobial therapy.

### 3.3 Hematological and Biochemical trends

The univariate analysis of blood count (**Table 3**) reveals that G3 has lower hemoglobin levels than G1, with a difference of 0.7 mg/dl (p=0.007). In terms of coagulation markers, G3 demonstrates higher D-dimer values than G1 (p=0.011). When examining the biochemistry results, the three groups displayed very similar levels of bilirubin. Additionally, G2 and G3 exhibited a median eGFR corresponding to stage 3 of the KDIGO 2012 scale, while G1 displayed levels corresponding to stage 2. Alkaline phosphatase was lower in G1 compared to G2, with a difference of less than 6 U/l (p=0.018), and compared to G3, with a difference of less than 16 U/l (p=0.006). Finally, potassium levels are slightly higher in G3 compared to G2 and G1.

**Table 3:** Result of the univariate analysis.

Characteristic	G1	G2	G3	p value	Corrected p value	N
Blood count ― Median (IQR)						
Absolute leucocytes ― x10^3/uL	6.00 (4.57;7.73)	6.90 (4.70;8.90)	7.10 (5.03;9.17)	**0.032**	0.092	294
Absolute neutrophils ― x10^3/uL	4.50 (3.20;5.90)	4.55 (3.22;6.56)	5.06 (3.39;7.09)	0.254	0.387	291
Absolute lymphocytes ― x10^3/uL	0.90 (0.60;1.20)	0.95 (0.67;1.37)	0.87 (0.60;1.21)	0.699	0.829	294
Haemoglobin ― mg/dl	13.6 (12.5;14.6)	13.2 (11.8;14.4)	12.9 (11.4;14.1)	**0.009**	**0.043**	296
Platelets ― x10^3/uL	168 (141;232)	170 (136;203)	187 (149;235)	0.168	0.299	292
Inflammation /infection markers ― Median (IQR)						
CRP ― mg/dl	44.8 (19.4;95.8)	59.4 (33.8;105)	42.0 (17.8;85.5)	0.096	0.219	287
Ferritin ― mg/dl	333 (170;660)	312 (138;717)	284 (135;598)	0.823	0.921	239
LDH ― U/l	609 (494;782)	509 (406;764)	536 (411;845)	0.068	0.167	269
Procalcitonin ― ng/ml	0.11 (0.08;0.20)	0.12 (0.08;0.24)	0.13 (0.07;0.26)	0.324	0.437	264
Coagulation Markers ― Median (IQR)						
D Dimer ― ng/ml	840 (526;1157)	970 (676;1446)	1099 (768;1645)	**0.012**	**0.046**	216
Fibrinogen ― mg/dl	709 (142)	704 (161)	696 (184)	0.875	0.921	249
Biochemistry and other markers ― Median (IQR)						
Glucose ― mg/dl	117 (106;134)	118 (105;140)	117 (101;144)	0.94	0.94	289
Total Bilirubin ― mg/dl	0.59 (0.43;0.78)	0.47 (0.36;0.68)	0.50 (0.32;0.65)	**0.013**	**0.046**	259
creatinine ― mg/dl	0.87 (0.75;1.07)	0.97 (0.73;1.23)	0.90 (0.76;1.19)	0.328	0.437	290
GFR ― ml/min	80.3 (60.0;90.0)	67.7 (49.7;89.7)	74.6 (44.6;84.5)	**0.016**	**0.05**	293
Urea ― mg/dl	41.0 (30.0;50.2)	42.0 (31.0;62.0)	46.0 (33.0;62.0)	0.119	0.244	291
SGPT ― U/l	38.0 (28.0;47.0)	36.0 (24.0;62.0)	36.0 (23.0;51.0)	0.596	0.763	259
SGOT ― U/l	29.0 (16.8;40.7)	23.8 (15.8;43.8)	20.6 (13.9;38.9)	0.122	0.244	264
Alkaline phosphatase ― U/l	69.5 (53.0;84.0)	76.0 (62.0;107)	85.5 (62.8;112)	**0.004**	**0.027**	269
Sodium ― mMol/l	137 (135;139)	137 (135;139)	137 (134;139)	0.857	0.921	292
Potassium ― mMol/l	4.27 (3.90;4.60)	4.27 (3.85;4.86)	4.50 (4.07;4.92)	**0.009**	**0.043**	287
Ultrasensitive troponin ― ng/ml	14.0 (9.00;24.5)	17.0 (10.0;23.0)	19.0 (10.8;29.2)	0.181	0.306	239

IQR, interquartile range; CRP, C-reactive protein; LDH, Lactate dehydrogenase; eGFR, estimated glomerular filtration rates; SGPT, serum glutamate pyruvate transaminase; SGOT serum glutamic-oxaloacetic transaminase. Bold values refers to values with a statistically significant p-value (p<0.05).

No statistically significant results were found in the multivariate analysis after performing the p value adjustment. However, when the p values were not adjusted G3 exhibited a higher absolute leukocyte count than G1 by 2.45 (0.69; 4.21) (p=0.003) and a higher count than G2 by 2.01 (0.23;3.78) (p=0.022). When analyzing hemoglobin levels, G3 presented a lower value of -1.12 mg/dl (IQR -1.21; 0.19) compared to G1 (p=0.001). The alkaline phosphatase was found to be higher in G2 compared to G1 with a difference of 14.98 (0.29;29.66) (p=0.044). Finally, the difference in potassium levels was maintained, with G3 having a higher value than G1 with a difference of 0.32 (0.09;0.54) (p=0.003).

## 4 Discussion

The hypothesis of our study was that the severity of the inflammatory response would decrease over time in relation to VOC, so that patients with the Omicron variant would show a lower elevation of inflammation biomarkers. However, we could not demonstrate that patients who required hospitalization for COVID-19 during the analyzed periods, with infections by different variants of SARS-CoV-2, presented a different inflammatory response. The values of CRP, PCT, LDH, or ferritin, biomarkers frequently used in this disease, were not different among the three groups. However, the dimer-D, which has also been considered as an inflammation marker in COVID-19 patients, was found to have a statistically significantly higher value in patients with the omicron variant. Nevertheless, when adjusting for age and severity, this significance disappears.

The severity of COVID-19 disease is partly determined by patients’ age (22, 23). In our series, although patients in the more recent variants were on average 10 years older, we could not find differences between the groups in terms of mortality or disease severity determined by the maximum score on the WHO scale. This may be influenced by higher vaccination rates in group 3 and better knowledge and management of the disease. However, lung involvement may have been more severe in group 1, as demonstrated by a higher proportion of bilateral pneumonia and greater use of anti-inflammatory drugs such as dexamethasone or tocilizumab. It is important to note that no changes were made to the COVID-19 management protocol at the hospital during the study period, and these differences could potentially be related to variability in decision making among treating physicians.

Throughout the pandemic, different specific laboratory markers have been used to help establish the risk of progression. There is abundant evidence in the literature that certain markers of inflammation, such as CRP, PCT, hematological markers (lymphocyte and neutrophil counts, D-dimer, ferritin), cardiac markers (troponin or CK-MB), or hepatic markers (AST, ALT, bilirubin), are useful in determining the prognosis of the disease (24). From the results of our study, we can confirm that patients who require hospitalization for COVID-19 present a similar inflammatory response regardless of the VOC responsible for the disease. Even the D-dimer, a recognized marker of thrombosis, is significantly higher in patients in group 3, with the Omicron VOC.

This absence of differences could be interpreted as indicating that the inflammatory response is dictated by the severity of the disease, not by the variant of virus causing it. If a patient suffers a severe enough infection to require hospitalization, the inflammatory response measured by commonly used biomarkers will be similar if the severity of the condition is similar.

Our study has some limitations. The assumption that it is a specific VOC is based on epidemiological data, not confirmation in the study patients. However, the appearance of a specific VOC is related to rapid expansion and widespread substitution of the previous variant by the new variant, so we believe that the periods analyzed reflect infections by different VOC. It is an observational, retrospective study with inevitable selection biases in this type of study. We selected consecutively hospitalized patients in the periods studied without any prior criteria, so we believe it is a selection with little bias. Nonetheless, the findings might be significant in broadening our understanding of the inflammatory reaction in individuals suffering from COVID-19.

In conclusion, the inflammatory response in hospitalized COVID-19 patients, determined by biomarkers such as CRP, ferritin, PCT or LDH, is similar between infections caused by different VOCs between 2021 and 2022. It appears that the severity of COVID-19 in hospitalized patients may determine the level of inflammatory response, rather than the particular viral strain responsible for the illness.

## Data availability statement

The raw data supporting the conclusions of this article will be made available by the authors, without undue reservation.

## Ethics statement

The studies involving humans were approved by García Arenillas. CEIm Hospital Clínico San Carlos. The studies were conducted in accordance with the local legislation and institutional requirements. Written informed consent for participation was not required from the participants or the participants’ legal guardians/next of kin because the study design does not pose major risks for the participants as it is an observational and retrospective study. On the other hand, taking into account the intention to include inpatients consecutively, the fact that some of the patients have died and that it is a retrospective study covering the period from 2021 to 2022, it is practically impossible to collect the informed consent of all the study subjects. Since these aspects are fundamental for obtaining results that are faithful to reality, the requirement of individual consent would make it impractical to carry out the study.

## Author contributions

J-RH-F: Conceptualization, Formal Analysis, Investigation, Methodology, Project administration, Supervision, Validation, Visualization, Writing – original draft, Writing – review & editing. AV: Conceptualization, Data curation, Formal Analysis, Investigation, Methodology, Validation, Writing – review & editing. AG: Investigation, Validation, Writing – review & editing. NC: Conceptualization, Validation, Writing – review & editing. IO: Investigation, Validation, Writing – review & editing. EO: Conceptualization, Validation, Writing – review & editing. CF: Investigation, Validation, Writing – review & editing. MM: Conceptualization, Validation, Writing – review & editing. VE: Conceptualization, Methodology, Validation, Writing – original draft, Writing – review & editing.

## HCSC infectious diseases group collaborators:

Rafael Sánchez-del-Hoyo: Hospital Clınico San Carlos, IdISSC, Unidad de Apoyo Metodologico a la Investigacion y departamento preventivo, Madrid, Spain. Isabel Zarza, Valeria Cabral, Nieves Sans, Javier Rodríguez-Añover: Servicio de Enfermedades Infecciosas, Instituto de Investigacion Sanitaria del Hospital Cınico San Carlos (IdISSC), Hospital Clínico San Carlos, Madrid, Spain.
